# Lightweight Machine Learning Framework for Cavitation Detection in Submersible Pumps Using Experimental Multi-Sensor Data

**DOI:** 10.3390/s26175596

**Published:** 2026-09-03

**Authors:** Seyit Alperen Celtek, Seyma Sattuf, Farhad Shahnia, Nuri Orhan

**Affiliations:** 1 Department of Electric Electronic Engineering, Karamanoglu Mehmetbey University, 70100 Karaman, Türkiye; 2 Department of Energy Systems Engineering, Karamanoglu Mehmetbey University, 70100 Karaman, Türkiye; 3 School of Engineering and Energy, Murdoch University, Perth, WA 6150, Australia; 4 Department of Agricultural Machinery and Technology Engineering, Selcuk University, 42100 Konya, Türkiye; nuriorhan@selcuk.edu.tr

**Keywords:** agricultural pumping systems, cavitation detection, machine learning, predictive maintenance, edge devices

## Abstract

Cavitation is one of the major factors reducing the performance, efficiency, and service life of deep well submersible pumps used in agricultural irrigation systems. Therefore, the early detection of cavitation is essential for improving pump reliability and reducing maintenance costs. In this study, a machine learning-based framework is proposed to detect and classify cavitation conditions using experimental data collected from a deep well pump test unit. Hydraulic and operational parameters were measured under different operating conditions, while the measured noise level was used only to assign cavitation labels during dataset preparation. According to the measured noise level, the operating conditions were classified into three categories: Normal, Incipient Cavitation, and Severe Cavitation. Several machine learning algorithms were evaluated using stratified cross-validation and an independent test dataset. Model performance was assessed using Accuracy, Precision, Recall, and F1-score. The results showed that the Extra Tree classifier achieved the best performance with an accuracy of 82.4%. Feature importance analysis indicated that power consumption and submergence depth were the most influential parameters for cavitation detection. Unlike many existing studies that rely on computationally intensive models, the proposed framework employs a simple and lightweight machine learning approach while maintaining reliable prediction performance. Its low computational complexity makes it a promising candidate for future implementation on resource-constrained edge devices, enabling real-time cavitation monitoring in agricultural pumping systems.

## 1. Introduction

Agricultural groundwater pumping systems play a critical role in irrigation management, crop productivity, and sustainable water resource utilization worldwide [1]. Submersible pumps are among the most widely used technologies for extracting groundwater in agricultural applications, particularly in regions where irrigation is essential for crop production [2]. Although pumps play a central role in agricultural water management and irrigation operations, their performance is frequently compromised by their challenging operating and environmental conditions. Current research shows that submersible pumps used in agricultural wells (farm bores) are particularly susceptible to failures caused by mechanical wear, electrical malfunctions, hydraulic instabilities, and adverse environmental conditions. These factors can reduce system reliability, increase maintenance costs, and cause unplanned shutdowns that negatively affect irrigation continuity and agricultural water supply systems [3,4].

Among these failure mechanisms, cavitation is one of the most critical hydraulic phenomena affecting the performance, efficiency, and operational lifetime of submersible pumps. Cavitation occurs when the local fluid pressure falls below the vapor pressure, causing vapor bubbles to form and subsequently collapse [5,6]. The collapse of these bubbles generates intense noise, vibration, and localized pressure fluctuations that may progressively damage pump components such as impellers and casings [7].

In agricultural groundwater pumping systems, cavitation is associated with insufficient submergence depth, excessive pumping rates, and unfavorable hydraulic conditions [8]. Consequently, the early detection of cavitation-related symptoms, such as abnormal noise generation and pressure variations, is essential for preventing mechanical damage and maintaining efficient pump operation.

Preventive maintenance is traditionally performed at predetermined service intervals regardless of the actual operating condition of the equipment. Although this strategy can reduce the probability of unexpected failures, it may also lead to unnecessary component replacement and increased maintenance costs [9]. To overcome these limitations, predictive maintenance has emerged as a more effective alternative which utilizes operational data collected from sensors to continuously monitor equipment condition and detect early signs of degradation [10]. By analyzing parameters such as vibration, noise, temperature, and pressure, predictive maintenance enables timely maintenance decisions before severe failures occur [11,12]. Recent advances in sensor technologies, data acquisition systems, and machine learning techniques have significantly improved the capabilities of predictive maintenance applications [13,14]. These technologies allow continuous monitoring of pump operating conditions and facilitate the rapid identification of abnormal behaviors associated with developing faults, including cavitation [15]. Recent advances in sensor technologies, wireless communication systems, and machine learning have enabled continuous monitoring of agricultural pumping systems and the collection of large volumes of operational data [16]. These developments have created new opportunities for the early detection of pump faults and cavitation-related abnormalities. However, cloud-based monitoring architectures still face several challenges, including communication latency, increased computational costs, and reduced reliability in environments with unstable network connectivity [17,18]. Such limitations may hinder the deployment of real-time monitoring systems, particularly in remote agricultural areas where communication infrastructure is often limited.

To address these challenges, edge computing has emerged as an effective alternative in which data processing is performed close to the data source rather than in centralized cloud servers. By reducing communication delays and network dependency, edge-based systems enable faster decision-making and improve the reliability of real-time monitoring applications [19,20].

Although significant progress has been achieved in predictive maintenance and machine learning applications for pump monitoring, several challenges remain. Many existing studies focus on fault detection under industrial operating conditions and often require high computational resources or extensive sensor infrastructures. Furthermore, most reported approaches have not been specifically developed for cavitation detection in agricultural pumping systems operating under varying hydraulic conditions. Consequently, there remains a need for reliable, computationally efficient, and practically deployable frameworks that can identify cavitation conditions using commonly measured pump parameters.

With the above backdrop, this study proposes an experimentally validated machine learning-based cavitation detection framework for agricultural pumping systems using real experimental data collected from a deep well pump test unit. Unlike many existing studies that primarily rely on vibration or acoustic measurements as direct model inputs, the proposed framework utilizes readily available hydraulic and operational parameters to classify cavitation conditions, thereby reducing sensing complexity while improving practical applicability. Unlike acoustic threshold-based approaches, the proposed framework estimates cavitation severity without requiring continuous acoustic measurements during operation, since acoustic data are used only during the offline dataset labeling stage. Rather than introducing a new machine learning algorithm, the novelty of this study lies in the development of a computationally efficient and experimentally validated framework that systematically compares multiple state-of-the-art machine learning classifiers under identical operating conditions and interprets their predictions in accordance with the physical mechanisms governing cavitation formation. By combining hydraulic measurements with data-driven classification techniques, the proposed approach enables reliable cavitation identification while maintaining low computational complexity and suitability for practical deployment in agricultural pumping systems. The main contributions of this study can be summarized as follows:

- An experimentally validated machine learning framework is developed for the detection and classification of Normal, Incipient, and Severe cavitation conditions in agricultural submersible deep well pumps.
- A real experimental dataset consisting of hydraulic and operational measurements collected under different operating conditions is established and utilized for model development and evaluation.
- A comprehensive comparison of eleven widely used machine learning algorithms is performed under identical experimental conditions to identify the most suitable classifier for cavitation detection.
- The physical significance of the selected hydraulic variables is investigated through correlation analysis and feature importance evaluation, linking the machine learning results with the underlying mechanisms of cavitation formation.
- The proposed framework demonstrates that reliable cavitation detection can be achieved using commonly measured hydraulic and operational parameters without requiring vibration measurements as model inputs, thereby improving its practical applicability for agricultural pumping systems.
- The developed framework is computationally efficient, experimentally validated, and suitable for future edge-based predictive maintenance applications in agricultural pumping systems.

The remainder of the paper is structured as follows. Section 2 presents a comprehensive review of the literature related to cavitation monitoring, predictive maintenance, and machine learning applications in pump systems. Section 3 describes experimental setup, dataset preparation, and machine learning methodology. Section 4 presents experimental results, performance evaluations, and physical validation analyses. Finally, Section 5 summarizes the main findings and discusses future research directions.

## 2. Literature Review

In recent years, machine learning-based predictive maintenance and fault diagnosis applications have gained considerable attention in the monitoring systems of pumps used in agricultural water management and irrigation infrastructures [21,22]. The increasing availability of sensor data and advances in data analytics have enabled the development of intelligent monitoring systems capable of identifying abnormal operating conditions before severe failures occur [23]. As a result, machine learning techniques have become important tools for improving the reliability, efficiency, and operational sustainability of agricultural pumping systems [24].

Among the various machine learning approaches reported in the literature, ensemble learning algorithms have demonstrated particularly promising results for pump monitoring and fault detection applications [25]. Methods such as Random Forest, Gradient Boosting, and Extreme Gradient Boosting (XGBoost) are capable of handling complex nonlinear relationships among operational variables while providing relatively high predictive accuracy and model interpretability [26]. These characteristics make ensemble learning techniques attractive for monitoring hydraulic systems where multiple operating parameters interact simultaneously.

Several studies have shown that ensemble learning models can successfully estimate pump performance parameters and detect early-stage failures using sensor measurements collected during operation [27,28]. Despite their strong predictive capabilities, these methods may be sensitive to data quality, feature selection, and hyperparameter settings. Consequently, model performance can deteriorate when sensor noise, missing data, or highly variable operating conditions are present, which are common characteristics of agricultural pumping systems [29,30].

In addition to ensemble learning methods, deep learning techniques have also been widely investigated for pump monitoring and fault diagnosis. In particular, the Long Short-Term Memory (LSTM) networks have demonstrated strong capabilities in capturing temporal dependencies and complex dynamic behaviors within pump operating data [31]. By learning sequential patterns from time-series measurements, these models can provide highly accurate predictions of abnormal operating conditions and equipment degradation.

Several machine learning models have been applied to cavitation monitoring in pumps. Orhan et al. developed ANN, SVM, and Decision Tree Regression models to predict NPSH, vibration, and noise levels under cavitation conditions, demonstrating that ANN achieved the highest prediction accuracy. However, their approach focused on predicting cavitation-related hydraulic parameters rather than directly classifying cavitation severity [32].

Deep learning has also shown promising performance for cavitation diagnosis. Yu and Cheng proposed a VMD-SWO-BiLSTM model based on pressure fluctuation signals for axial-flow pumps, achieving high identification accuracy. Nevertheless, the method requires complex signal processing and computationally intensive optimization, limiting its applicability in lightweight monitoring systems [33].

Recent reviews indicate that most machine learning studies rely on laboratory-scale datasets, vibration or pressure signals, and computationally demanding deep learning models. Consequently, lightweight and interpretable machine learning frameworks using routinely measured operational parameters remain relatively limited [34].

Overall, although considerable progress has been achieved in machine learning-based pump monitoring and fault diagnosis, several important challenges remain unresolved. Existing approaches are often sensitive to data quality, exhibit limited robustness under noisy and variable operating conditions, and are rarely validated using real experimental datasets collected from agricultural pumping systems. In addition, many high-performance models require substantial computational resources, limiting their practical deployment in real-time monitoring applications.

Therefore, there remains a clear need for a computationally efficient and reliable machine learning framework capable of accurately detecting cavitation using commonly measured hydraulic and operational parameters. Motivated by these challenges, the present study proposes a lightweight cavitation detection framework for agricultural pumping systems based on experimental sensor data and comparative machine learning analysis. The proposed approach aims to provide an effective balance between prediction accuracy, computational efficiency, and practical applicability for real-world irrigation systems.

## 3. Methodology

### 3.1. Experimental Setup

**Figure 1:**
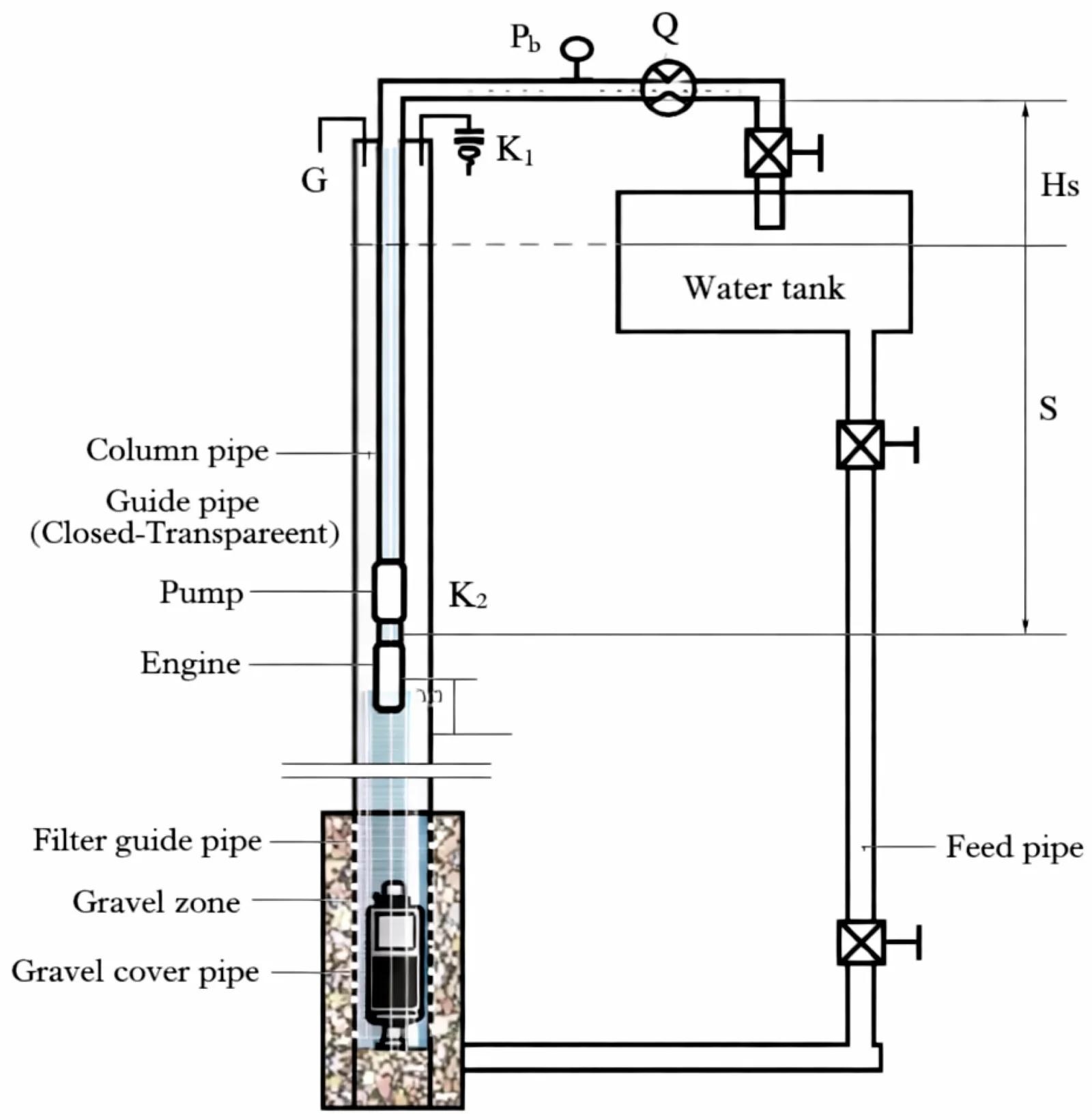
Experimental setup of the submersible deep well pump test system.

The dataset used in this study was obtained from controlled experimental measurements carried out at the deep well pump test facility located in the Department of Agricultural Machinery and Technology Engineering, Faculty of Agriculture, Selçuk University, Konya, Turkey. The experimental infrastructure is specifically designed to simulate the real operating conditions of agricultural groundwater pumping systems as shown in Figure 1.

The experimental setup consists of a vertical deep well system with a total height of approximately 10 m, containing different structural sections to simulate realistic subsurface conditions. The lower part of the system includes a well strainer section surrounded by gravel material with a controlled particle size distribution, allowing for representation of natural filtration and flow conditions. Above this section, a transparent pipe segment allows for visual observation of flow behavior, including vortex formation and cavitation events. The upper part of the system is completed with a steel casing structure to ensure mechanical stability.

During the experiments, a submersible deep well pump driven by an electric motor with a nominal power of 4 kW was used. The pump was operated under various flow rate conditions, and its performance was evaluated by systematically adjusting the hydraulic conditions within the test system. Specifically, the water level in the well was gradually lowered to simulate different submersion levels, a critical parameter affecting pump performance and cavitation formation. The submersion depth (S) was calculated based on the difference between the initial water level and the drop (Δ), representing the dynamic response of the system during operation.

A comprehensive sensor system was employed to measure key operational parameters of the pump system. These measurements include flow rate, pressure, power consumption, noise level, and water level variations. The flow rate was measured using an electromagnetic flow meter with high accuracy, while pressure values were obtained through industrial-grade manometers. Noise levels, which are particularly important for cavitation detection, were monitored using a Lutron PCE-SLT digital sound-level indicator coupled with a TR-SLT1A4 acoustic probe. The probe provides selectable measurement ranges of 30–80, 50–100, and 80–130 dB and generates a 4–20 mA output signal. The indicator has a specified accuracy of ±(0.15% + 2 digits) and a response time of 1 s. The acoustic probe was installed adjacent to the transparent well section and positioned close to the pump inlet region so that changes associated with vortex development and air ingestion could be detected consistently. Additionally, temperature and velocity measurements were recorded to support a more comprehensive analysis of pump behavior. All measurement devices were selected to comply with relevant international standards (EN ISO 3740 [35] and EN ISO 9906 [36]), ensuring the reliability and repeatability of the experimental data.

To capture the dynamic behavior of the system, an automated data acquisition system was developed. Sensor data were transmitted to a central processing unit via a wireless communication interface and recorded at predefined time intervals. During each experimental condition, multiple measurements were collected and averaged to reduce noise and improve data consistency. This approach enabled the creation of a structured and reliable dataset representing different operating regimes of the pump system.

The experiments were conducted under multiple flow rate conditions, typically ranging between 40 and 60 m^3^/h, and for each flow condition, measurements were performed at several submergence levels. As the water level decreased, the system was re-adjusted to maintain the desired flow rate using control valves. This procedure allowed the collection of data across a wide range of operating conditions, including those leading to vortex formation and cavitation.

### 3.2. Dataset Description

The dataset used in this study consists of experimental measurements obtained under controlled operating conditions of a submersible deep well pump system. Each data sample represents the steady-state operating point of the pump under a specific flow rate and submersion level. The dataset includes the fundamental hydraulic, mechanical, and acoustic variables that characterize the operational behavior of the system.

The primary variables considered in the dataset are flow rate (Q), submersion depth (S), drop (Δ), power consumption (N), discharge pressure (Pb), and noise level (G). These parameters were selected due to their direct relationships with pump performance and their sensitivity to anomalous operating conditions such as cavitation. In particular, noise level measurements play a critical role as an indirect indicator of cavitation intensity.

Before applying machine learning models, the preprocessing steps were performed to ensure data consistency, reliability, and suitability for analysis. First, the raw data obtained from the experimental system were examined for inconsistencies such as irregular column structures, non-numeric inputs, and formatting differences arising from measuring instruments and recording software. Column names were standardized, and a clean and structured dataset was created by removing unnecessary or empty columns. Then, invalid or unrepresentative data points were filtered out. Rows containing missing values or incomplete measurements were removed from the dataset. As a result of these preprocessing steps, a refined dataset with improved consistency and reduced noise was obtained.

The final processed dataset consisted of 82 steady-state operating samples collected under controlled laboratory conditions. Based on the acoustic classification strategy described in Section 3.3, the dataset comprised 26 Normal, 41 Incipient Cavitation, and 15 Severe Cavitation samples. Measurements were collected under multiple combinations of flow rate and submergence depth. For each operating condition, multiple measurements were performed and subsequently averaged to reduce measurement variability and improve data consistency. This procedure enhanced the repeatability of the experimental data while providing representative operating points covering a broad range of hydraulic conditions relevant to cavitation development. Since the dataset consists of steady-state experimental operating points rather than continuous time-series measurements, a fixed temporal sampling interval is not applicable. For each operating condition, multiple steady-state measurements were collected after the hydraulic variables stabilized and subsequently averaged to obtain a representative sample.

### 3.3. Cavitation Classification Strategy

In this study, cavitation conditions in the pump system were defined and classified according to the measured noise level (G), which is widely accepted as an indirect but reliable indicator of cavitation events in hydraulic systems [37]. As cavitation intensity increases, the formation and collapse of vapor bubbles lead to a significant increase in noise levels, generating characteristic acoustic signals. The measured acoustic response was therefore adopted as the primary variable for assigning cavitation labels during dataset preparation.

Based on experimental observations and the distribution of measured noise data, a threshold-based classification strategy was adopted to separate the operating conditions into three different cavitation states, i.e., Normal, Initial, and Severe cavitation, defined as follows:

- Normal condition (Normal): G<73 dBA
- Incipient cavitation (Incipient): 73≤G<78 dBA
- Severe cavitation (Severe): G≥78 dBA

**Figure 2:**
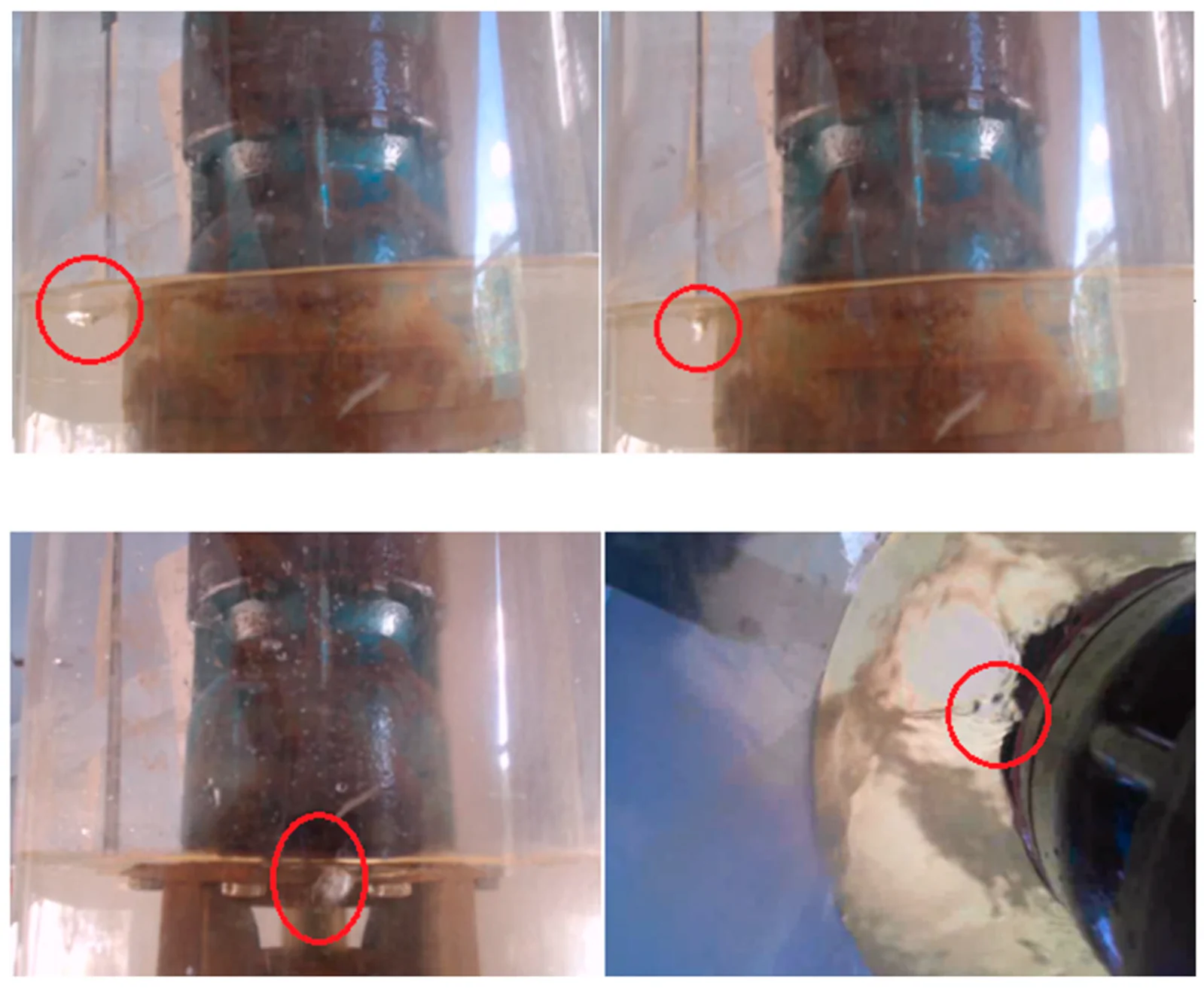
Visual observations used to support the acoustic classification: (**top**) vortex formation without air ingestion into the pump, associated with comparatively lower noise levels; and (**bottom**) vortex extension to the pump inlet and visible air ingestion, associated with increased acoustic emissions.

These threshold values were selected according to the observed transition regions in the experimental dataset where significant changes in acoustic behavior and flow stability were detected. These threshold values were not selected solely as statistical divisions of the dataset but were established according to experimentally observed transition regions in the measured acoustic response. During the experiments, the water level was gradually lowered while the pump inlet region was simultaneously monitored through the transparent section of the test facility (Figure 2). The acoustic measurements were evaluated together with the corresponding visual observations of the inlet-flow behavior.

Under sufficiently submerged operating conditions, no persistent free-surface vortex extending toward the pump inlet was observed, and the measured noise remained within the normal operating range. As the submergence depth decreased, a free-surface vortex gradually developed. During this transitional stage, the vortex core remained separated from the pump inlet, no visible air ingestion occurred, and the acoustic level increased moderately. This operating region was classified as Incipient Cavitation. At lower submergence depths, the vortex extended to the pump inlet and visible air ingestion occurred. This hydraulic transition was accompanied by a pronounced increase in the measured acoustic level and was therefore classified as Severe Cavitation.

Specifically, the mid-range (73–78 dBA) represents the onset of cavitation, where early-stage bubble formation begins to affect system performance. Beyond this threshold, higher noise levels indicate concentrated cavitation, which can lead to performance degradation and potential mechanical damage. Accordingly, the 73 dBA threshold represents the experimentally observed transition from stable inlet-flow behavior to persistent vortex development, whereas the 78 dBA threshold corresponds to the onset of vortex-induced air ingestion accompanied by substantially increased acoustic emissions. Therefore, the adopted thresholds reflect experimentally observed hydraulic transitions rather than purely statistical partitions of the dataset.

It should be noted that the measured noise level was used exclusively for assigning cavitation labels during dataset preparation and was not included as an input feature for any machine learning model. The models were trained and evaluated solely using the selected hydraulic and operational parameters described in Section 3.4, thereby avoiding potential data leakage between the labeling process and the model inputs.

This labeling approach transforms the problem into a multi-class classification task, enabling the application of supervised machine learning algorithms to automatically identify cavitation conditions based on input characteristics. Although the cavitation labels were primarily based on acoustic measurements, they were additionally supported by visual observations of vortex development and air ingestion obtained during the experiments. The use of noise-based labeling also provides a practical advantage, as acoustic measurements can be obtained in real time and without intrusive instrumentation, making the proposed approach suitable for real-world applications.

It should be noted that the cavitation classification thresholds are derived from experimental observations in this specific setup. As such, these thresholds may vary depending on pump design, operating conditions, and measurement configuration. The use of a single acoustic indicator may also introduce uncertainty in distinguishing cavitation stages. Direct vibration measurements and high-frequency suction-pressure fluctuation measurements were not available in the present study. Therefore, the proposed operating classes should be interpreted as experimentally defined cavitation states supported by acoustic and visual evidence, rather than direct measurements of vapor volume fraction or cavitation erosion intensity.

Furthermore, the adopted acoustic thresholds were established specifically for the experimental pump and test conditions considered in this study. Consequently, these threshold values should not be interpreted as universally applicable limits but rather as experimentally defined criteria for dataset labeling. Future studies should determine appropriate threshold values for different pump designs, operating conditions, and installation environments.

### 3.4. Feature Selection and Input Configurations

To evaluate the impact of different input variables on the performance of the proposed cavitation detection technique, two distinct feature sets were defined and analyzed in this study. The selection of input variables was based on both physical relevance to pump operation and their potential contribution to cavitation-related behavior.

The first feature set (Input Set 1) includes the following variables: flow rate (Q), submergence depth (S), drawdown (Δ), power consumption (N), and discharge pressure (Pb). This set represents a comprehensive configuration that incorporates both hydraulic and operational parameters. In particular, drawdown (Δ) is included as it reflects the dynamic change in water level during pump operation and is directly related to submergence conditions, which are known to influence cavitation formation.

The second feature set (Input Set 2) is defined by excluding the drawdown parameter (Δ), and includes flow rate (Q), submergence depth (S), power consumption (N), and discharge pressure (Pb). The primary motivation for this reduced configuration is to investigate whether comparable predictive performance can be achieved with fewer input variables. This is particularly important for practical applications, where reducing the number of required sensors can decrease system complexity, cost, and maintenance requirements.

By comparing these two configurations, the study aims to analyze the trade-off between model complexity and prediction performance. While Input Set 1 provides a more detailed representation of system dynamics, Input Set 2 offers a simplified and potentially more deployable solution, especially for edge-based predictive maintenance systems. From a practical perspective, the two input configurations also represent different levels of measurability and applicability in real-world agricultural systems. Input Set 1 includes parameters such as drawdown (Δ), which are directly related to the physical mechanisms of cavitation formation but are relatively difficult to measure continuously in field conditions due to additional instrumentation requirements. In contrast, Input Set 2 consists of commonly measured operational variables such as flow rate, pressure, and power consumption, which are already available in most irrigation pump systems. Therefore, Input Set 2 represents a more practical and deployable configuration for real-world applications. The comparison between these two input sets not only evaluates model performance but also investigates the feasibility of implementing predictive maintenance systems under real operational constraints.

### 3.5. Machine Learning Workflow and Evaluation Procedure

**Figure 3:**
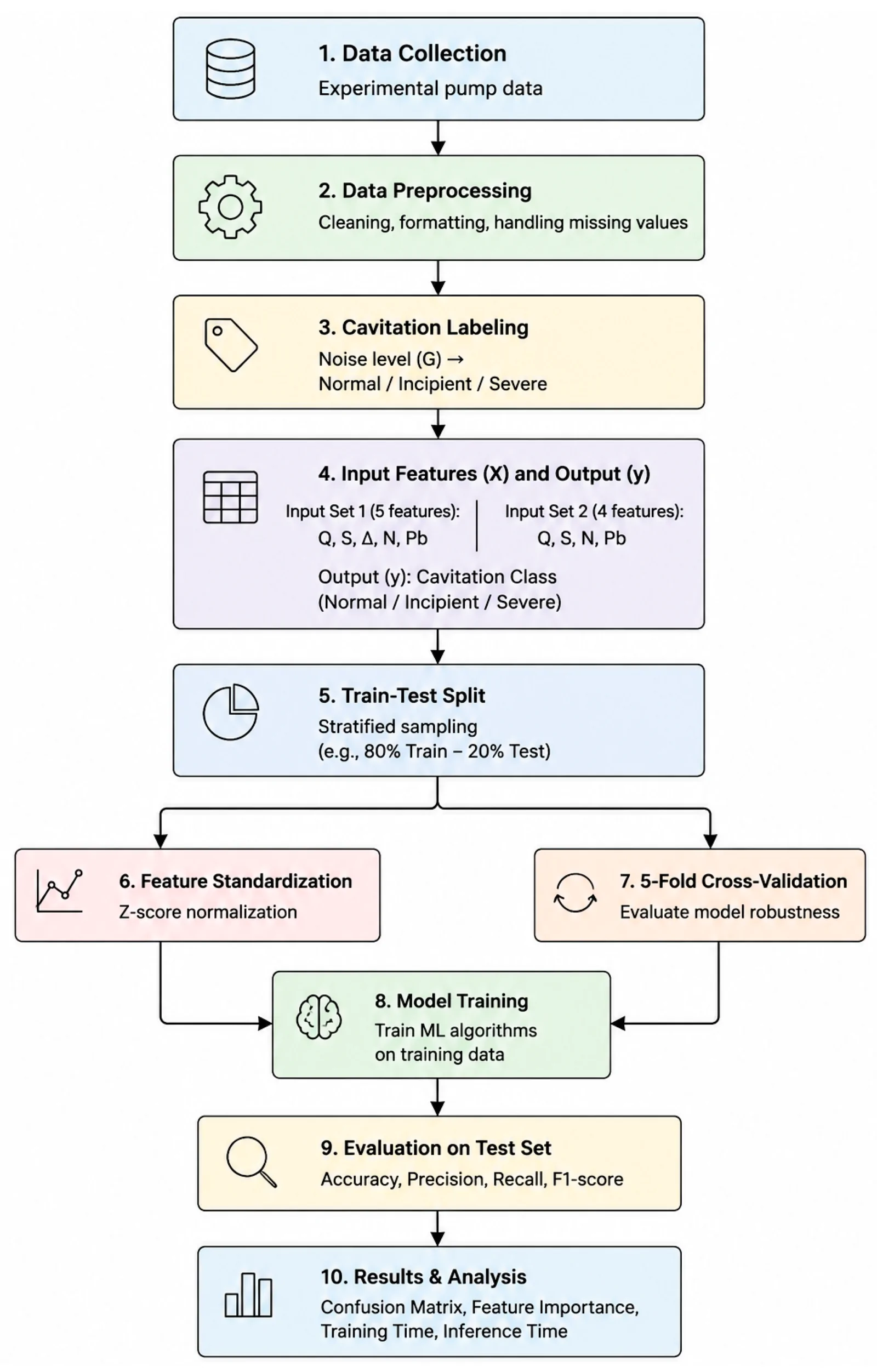
Proposed machine learning framework for cavitation classification.

The overall machine learning workflow employed in this study is illustrated in Figure 3. Following data preprocessing and cavitation labeling, the dataset was divided into input features and target classes. The selected input variables consisted of hydraulic and operational parameters measured during pump operation, whereas the target variable represented the cavitation condition.

Two different input configurations of Input Set 1 and Input Set 2 were evaluated, as introduced in Section 3.4. The output variable was defined as the cavitation state derived from the measured noise level (G). Based on the classification strategy described in Section 3.3, each sample was assigned to one of three classes: Normal, Incipient, or Severe cavitation. Consequently, the problem was formulated as a multi-class classification task, where the machine learning models aimed to predict the cavitation condition using only the selected hydraulic and operational parameters.

To evaluate the predictive capability of different machine learning algorithms, the dataset was randomly divided into training and testing subsets. Stratified sampling was employed to preserve the class distribution within both subsets.

Prior to model training, feature standardization was applied using z-score normalization. To ensure a consistent and unbiased evaluation procedure, the complete machine learning workflow followed a sequential pipeline. After cavitation labeling, the dataset was first divided into 80% training and 20% testing subsets using stratified sampling to preserve the class distribution. Each averaged operating condition was treated as a single independent sample. Therefore, no repeated measurements from the same operating condition appeared simultaneously in both the training and testing datasets. Feature standardization was then performed by computing the normalization parameters exclusively from the training data, and the same transformation was subsequently applied to the validation and test subsets. Five-fold stratified cross-validation was carried out only on the training data during model development, while the independent test dataset remained completely isolated until the final evaluation stage. This procedure minimized the risk of information leakage and ensured an unbiased assessment of model generalization performance.

Each machine learning algorithm was trained using the training dataset and subsequently evaluated using the unseen test dataset. In addition to the train–test evaluation, a five-fold stratified cross-validation procedure was performed to assess model robustness and reduce the influence of random data partitioning. During this process, the dataset was divided into five subsets, where four subsets were used for training and one subset was used for validation. This procedure was repeated five times, and the average performance was reported.

All machine learning models were implemented using the Scikit-learn library. The model-specific hyperparameter settings used throughout the experiments are summarized in Table 1. The evaluated algorithms were configured using either the default Scikit-learn parameters or user-defined settings when explicitly specified (e.g., the number of estimators or maximum number of iterations). For algorithms in which a parameter was not explicitly specified, the default values provided by the Scikit-learn implementation were retained. To ensure the reproducibility of the experimental results, a fixed random seed (random_state = 42) was used for data partitioning and for all algorithms involving stochastic processes.

Model performance was evaluated using Accuracy, Precision, Recall, and F1-score metrics. Accuracy represents the overall classification success, while Precision and Recall provide information regarding the reliability of positive predictions and the capability of detecting cavitation conditions, respectively. The F1-score was used as a balanced indicator combining Precision and Recall.

All machine learning models were developed and evaluated using Python 3.11 with the Scikit-learn library on a workstation equipped with an Intel Core i7 processor, 32 GB RAM, and the Windows 11 operating system.

## 4. Experimental Results

This section presents experimental results and evaluates the performance of the developed machine learning models. Correlation analysis and model performance comparisons are conducted to assess the effectiveness of the proposed framework.

**Figure 4:**
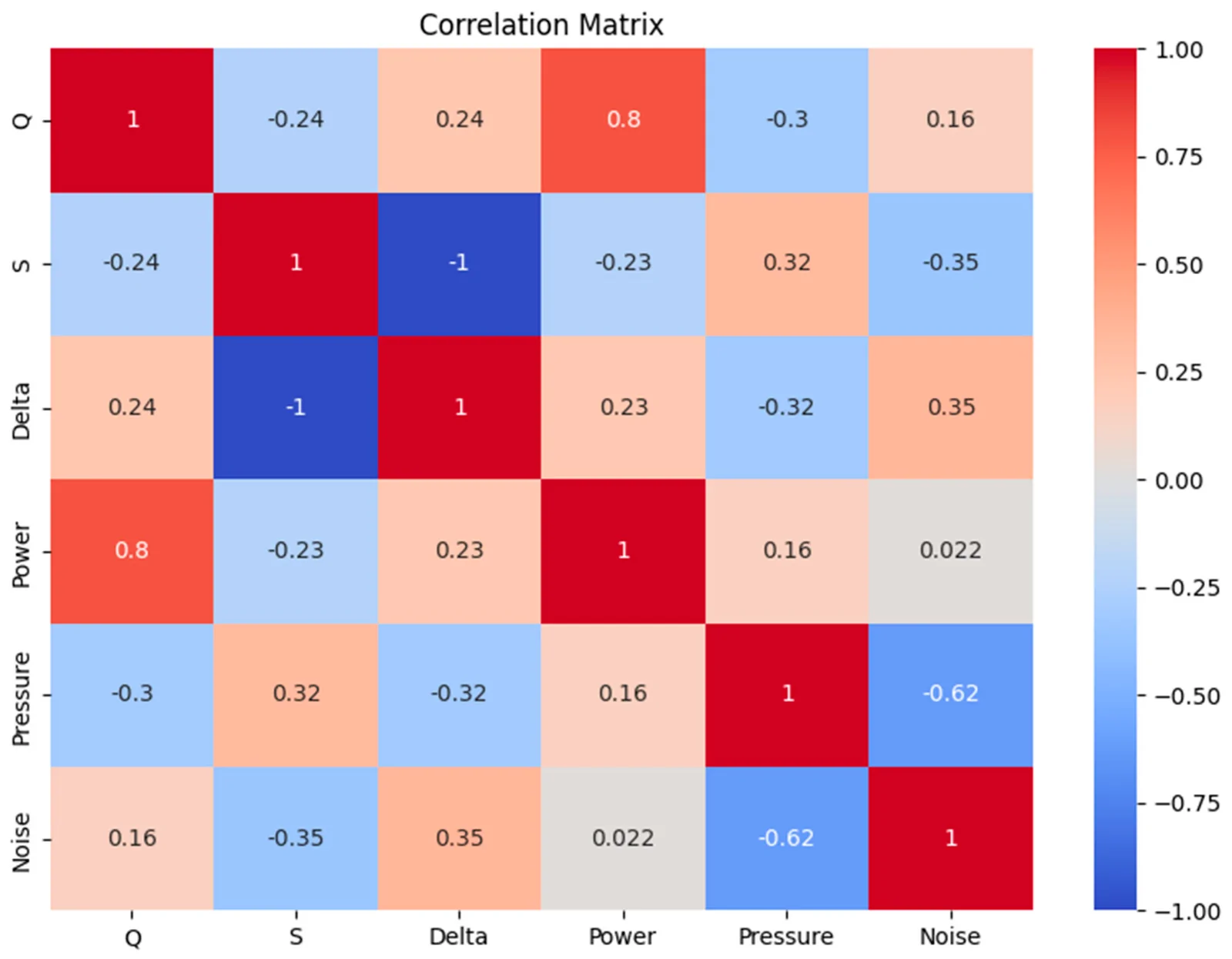
Correlation matrix of the selected input variables and noise level.

The correlation matrix of the selected variables is presented in Figure 4. Several meaningful relationships can be observed among the hydraulic, operational, and cavitation-related parameters. The strongest positive correlation was found between flow rate (Q) and power consumption (N) (r = 0.80), indicating that higher flow rates require greater energy consumption. As expected, submergence depth (S) and drawdown (Δ) exhibited a nearly perfect inverse relationship (r = −1.00).

The noise level (G), which was used for cavitation classification, showed its strongest correlation with discharge pressure (Pb) (r = −0.62). In addition, noise was moderately correlated with submergence depth (r = −0.35) and drawdown (r = 0.35), suggesting that cavitation becomes more pronounced as water level conditions deteriorate. Overall, the results indicate that pressure and water-level-related parameters play a significant role in cavitation formation and are therefore suitable inputs for machine learning-based cavitation detection.

**Figure 5:**
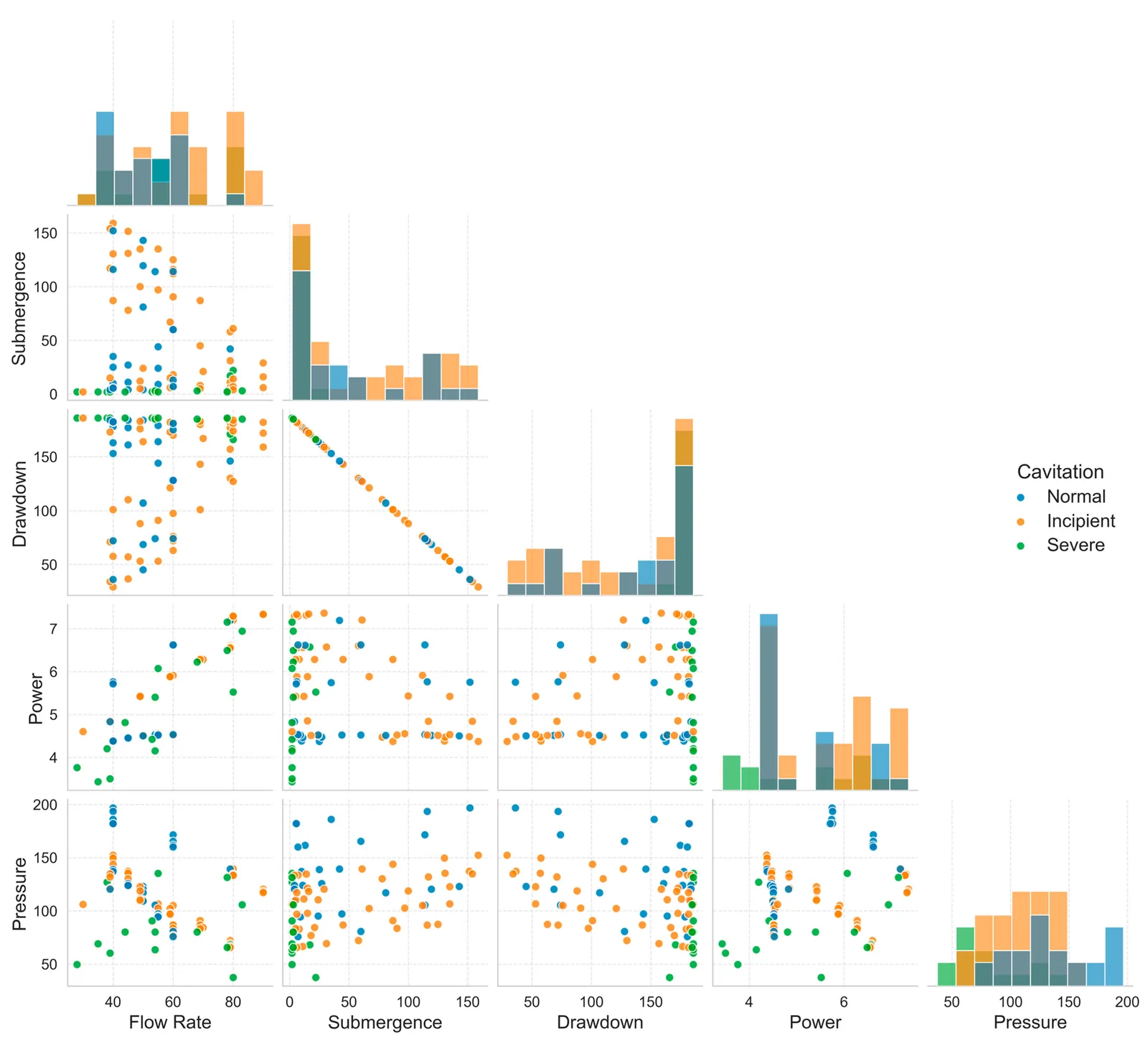
Pairwise visualization of the selected hydraulic and operational feature space used for cavitation classification.

To further illustrate the distribution of the selected hydraulic and operational variables, Figure 5 presents a pairwise visualization of the feature space used for cavitation classification. The scatter plots illustrate the relationships between different feature pairs, while the diagonal histograms show the distribution of each individual feature for the Normal, Incipient, and Severe Cavitation classes. The figure shows that no single feature completely separates the three cavitation classes. Considerable overlaps exist between the Normal and Incipient Cavitation classes, whereas the Severe Cavitation class becomes more distinguishable when multiple hydraulic variables are considered jointly. In particular, combinations of pressure, power consumption, and submergence depth exhibit improved class separability compared with any individual feature. These observations further support the use of multivariate machine learning models capable of capturing the nonlinear relationships among the selected input features.

The performance comparison of the evaluated machine learning models is presented in Table 2. The results indicate that tree-based algorithms generally outperformed the remaining classifiers in cavitation detection. Among all evaluated models, the Extra Tree classifier achieved the best overall performance, obtaining the highest cross-validation accuracy (84.8%) as well as the highest test accuracy (82.4%). It also yielded a precision of 85.0%, a recall of 82.4%, and an F1-score of 82.2%, demonstrating superior generalization capability for unseen cavitation data.

Random Forest, Decision Tree, Extra Trees, and Bagging also achieved competitive performance, with test accuracies ranging from 76.5% to 76.5%. These results indicate that tree-based learning methods are particularly effective in capturing the nonlinear relationships between hydraulic operating conditions and cavitation behavior. Among these models, Random Forest achieved the second-highest cross-validation accuracy (81.8%), although its final test performance remained below that of the Extra Tree classifier.

To further benchmark the proposed framework, Support Vector Machine (SVM with RBF kernel) and Multi-Layer Perceptron (MLP) classifiers were also evaluated using the same preprocessing pipeline and validation strategy. The SVM achieved a cross-validation accuracy of 78.0% and a test accuracy of 76.3%, demonstrating competitive performance but remaining below the best-performing tree-based models. In contrast, the MLP achieved a cross-validation accuracy of 65.8% and a test accuracy of 47.6%, indicating that the neural network-based approach was less effective for the relatively small experimental dataset considered in this study.

Gradient Boosting and Logistic Regression exhibited moderate performance, achieving test accuracies of 70.6%. In contrast, KNN, Naïve Bayes, HistGradientBoosting, and AdaBoost produced comparatively lower classification performance. The weakest results were obtained by AdaBoost, which achieved a test accuracy of only 41.2%, indicating that this boosting approach was less suitable for the characteristics of the present dataset.

Overall, the results demonstrate that tree-based machine learning algorithms consistently outperformed distance-based, probabilistic, linear, and boosting-based approaches for cavitation classification using hydraulic and operational pump parameters. Nevertheless, although the proposed framework achieved satisfactory classification performance, the obtained accuracy suggests that further improvements are possible, particularly in distinguishing between Incipient and Severe cavitation stages.

Table 3 compares the proposed framework with representative machine learning-based cavitation detection studies reported in the literature. Although several previous studies reported higher classification accuracies, most of them relied on vibration signals together with computationally intensive feature extraction techniques or deep learning models. In contrast, the proposed framework utilizes commonly measured hydraulic and operational parameters without requiring vibration signals as model inputs, thereby reducing sensing complexity while maintaining satisfactory classification performance.

To further evaluate the practical deployment potential of the proposed framework, an additional experiment was conducted by excluding the drawdown (Δ) parameter from the input feature set. This comparison was intended to assess the trade-off between classification performance and sensor requirements in practical pumping systems. The corresponding results are presented in Table 4.

As shown in Table 4, excluding the drawdown (Δ) parameter reduced the cross-validation accuracy from 84.8% to 81.7%, while the independent test accuracy decreased from 82.4% to 74.0%. These results indicate that drawdown provides complementary information for distinguishing different cavitation conditions and improves the overall classification performance of the proposed framework. Nevertheless, the model trained without the drawdown parameter maintained a reasonable level of predictive performance using only the remaining hydraulic and operational variables. This comparison demonstrates the trade-off between classification accuracy and sensor requirements. While including drawdown yields the highest predictive performance, excluding this parameter reduces sensing complexity and may be preferable in applications where drawdown measurements are unavailable or additional instrumentation is undesirable.

Table 5 presents the class-wise performance of the best-performing Extra Tree classifier. The model achieved high precision and recall for the Normal and Severe Cavitation classes, indicating reliable identification of these operating conditions. In contrast, the Incipient Cavitation class exhibited lower precision (77%), recall (73%), and F1-score (75%), suggesting that this intermediate stage is more challenging to distinguish due to the overlap of its characteristics with the adjacent cavitation states.

Table 6 presents the training times of the evaluated machine learning models. Considerable differences were observed among the algorithms in terms of computational requirements. Single-tree approaches such as Extra Tree and Decision Tree exhibited the shortest training times, whereas ensemble-based methods including Bagging, Random Forest, and Gradient Boosting required relatively longer training durations due to the construction of multiple decision trees. Nevertheless, the average training time across all evaluated models was only 0.052 s, indicating a very low computational burden. This result demonstrates that the proposed machine learning framework can be trained rapidly and updated efficiently when new data becomes available. Furthermore, the overall computational cost suggests that real-time cavitation monitoring and predictive maintenance applications can be implemented without significant processing limitations, even on resource-constrained edge devices commonly used in agricultural pumping systems.

To further investigate whether dimensionality reduction could provide a more compact representation of the input space, the principal component analysis (PCA) and linear discriminant analysis (LDA), both linear techniques for reducing the number of variables in a dataset [42], were additionally employed for the five selected hydraulic and operational variables. Since the proposed framework already employs a limited number of physically interpretable input features, PCA resulted in a further reduction in the feature space and led to a noticeable decrease in classification performance. LDA was also evaluated using the 82 experimental samples and provided a two-dimensional discriminant representation of the three cavitation classes. However, the direct classification accuracy obtained using the LDA representation was approximately 70.7%, compared with 82.4% obtained by the Extra Tree classifier using the original feature representation. Based on this observation, dimensionality reduction was not incorporated into the final classification pipeline in this study. This analysis confirms that retaining the original hydraulic and operational variables provides a more favorable balance between physical interpretability and classification performance in the case of this study.

**Figure 6:**
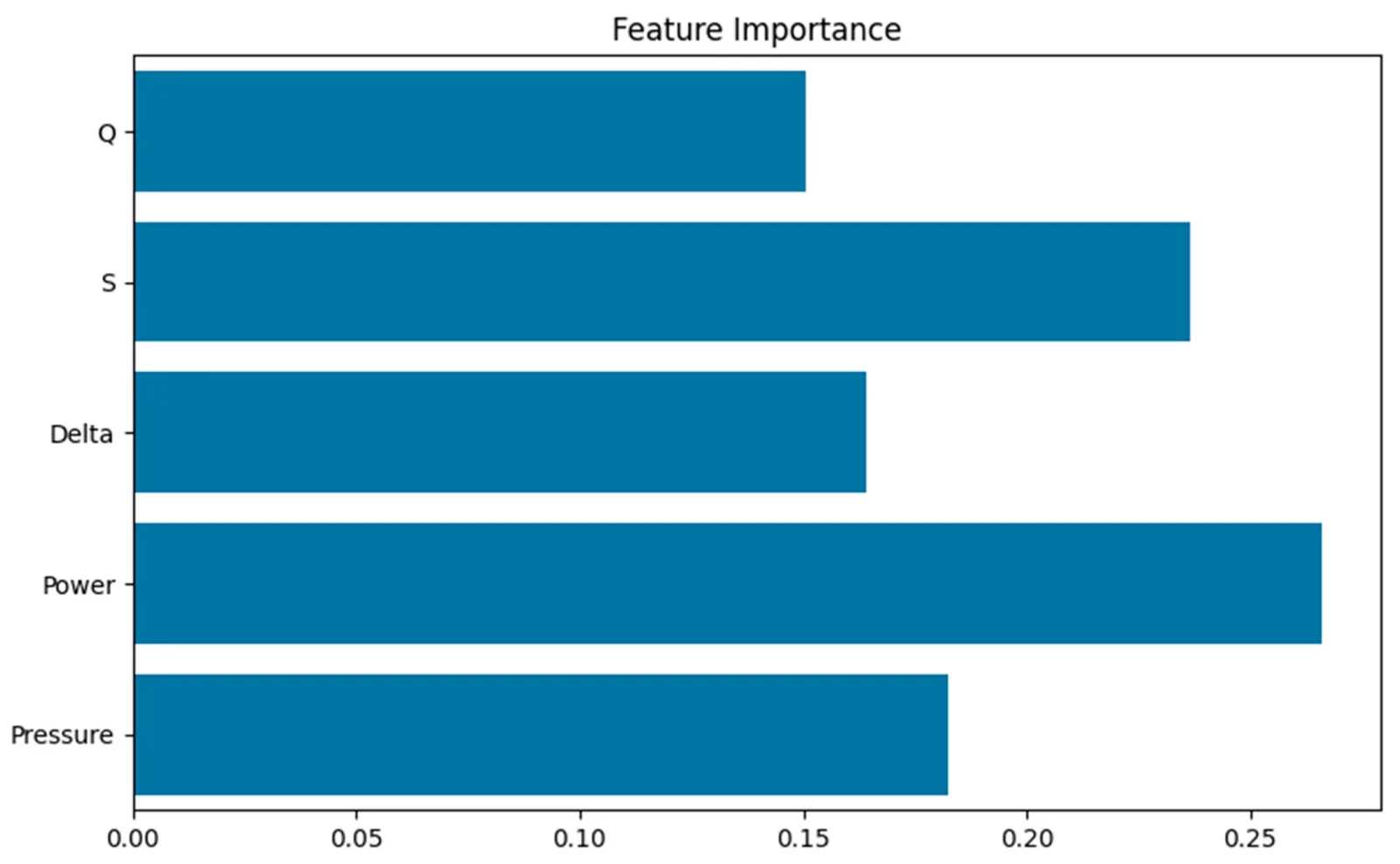
Feature importance values obtained from the Extra Tree classifier.

The feature importance results obtained from the best-performing Extra Tree classifier are presented in Figure 6. This analysis provides valuable insight into the relative contribution of each input variable to cavitation classification. Among the evaluated parameters, power consumption (N) was identified as the most influential feature, accounting for approximately 27% of the total importance. This finding suggests that variations in the energy demand of the pump are strongly associated with changes in cavitation conditions. As cavitation develops, vapor bubble formation and collapse disturb the hydraulic flow, reducing the hydraulic efficiency of the pump. Consequently, the pump motor experiences variations in power demand while compensating for the degraded hydraulic performance, explaining the high predictive importance of power consumption.

The second most important variable was submergence depth (S), with an importance value of approximately 24%. This result is physically meaningful because insufficient submergence is one of the primary causes of vortex formation and cavitation in deep well pumping systems. As the water level decreases and the available water column above the pump becomes smaller, the probability of cavitation increases. A lower water level also reduces the hydrostatic pressure at the pump inlet, thereby decreasing the available Net Positive Suction Head (NPSH). When the available NPSH approaches the required NPSH of the pump, vapor bubbles begin to form, initiating cavitation.

Pressure (Pb), drawdown (Δ), and flow rate (Q) also contributed to the classification process, although their relative importance was lower. Pressure exhibited an importance value of approximately 18%, indicating its relevance in representing hydraulic operating conditions. Pressure variations are closely associated with the unstable flow conditions created by bubble formation and collapse during cavitation, making pressure an effective indicator of cavitation severity. Similarly, drawdown and flow rate contributed approximately 16% and 15%, respectively.

Overall, the feature importance analysis demonstrates that cavitation formation is governed by a combination of hydraulic and operational parameters rather than a single variable. The agreement between the identified feature importance rankings and the established hydraulic mechanisms of cavitation indicates that the proposed machine learning framework captures physically meaningful relationships rather than merely statistical correlations. The results further indicate that power consumption and submergence depth are the most informative indicators for cavitation detection within the experimental conditions considered in this study.

## 5. Physical Validation of the Proposed Framework

**Figure 7:**
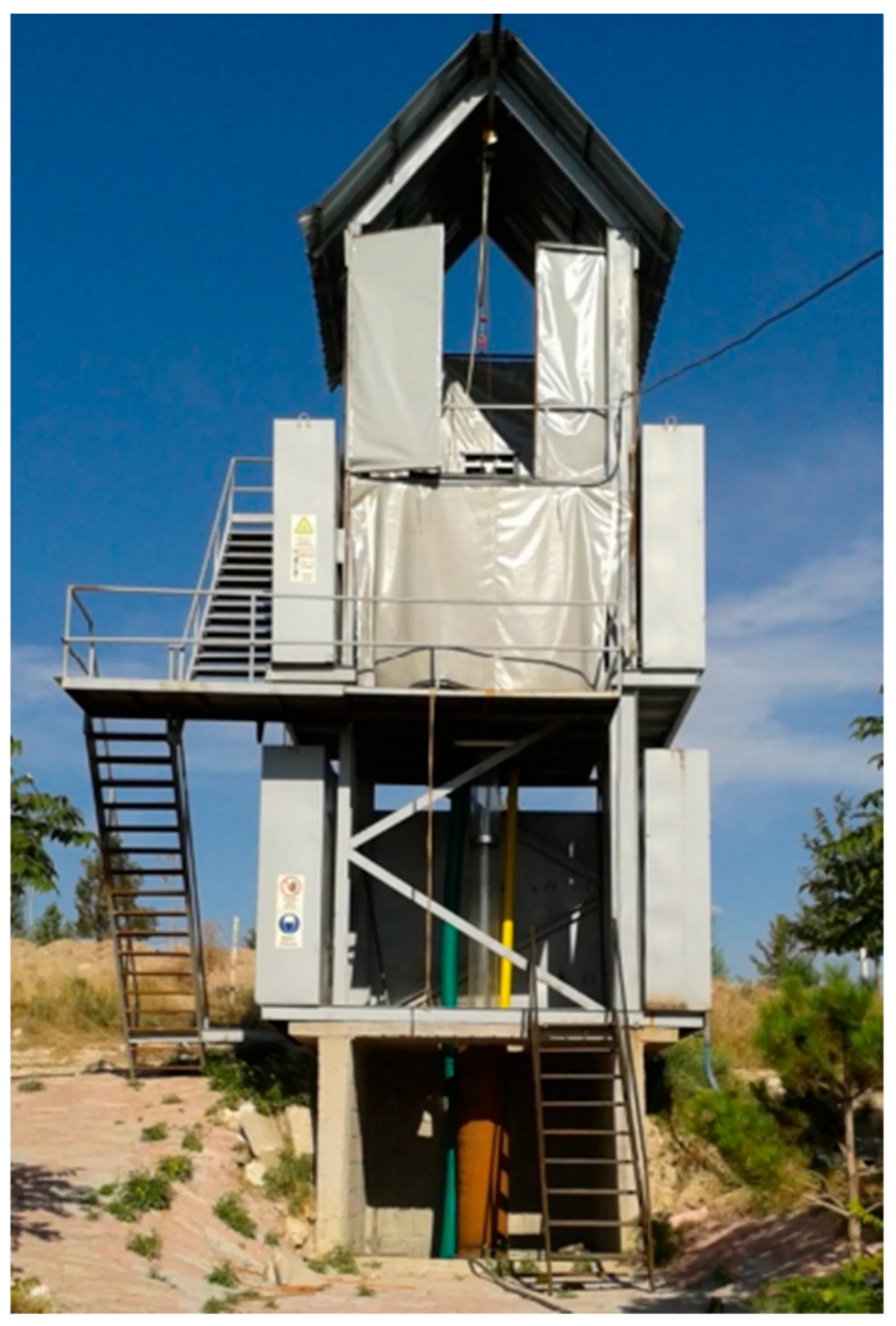
Deep Well Pump Test Unit used for experimental data collection at the Şinasi Yetkin Agricultural Machinery Application Workshop, Department of Agricultural Machinery and Technologies Engineering, Faculty of Agriculture, Selçuk University, Konya, Turkey.

To evaluate the practical applicability of the proposed framework, the best-performing Extra Tree classifier was tested using experimental data collected from the deep well pump test unit. Figure 7 presents the deep well pump test unit used for data collection. The experiments were conducted at the Deep Well Pump Test Unit established at the Şinasi Yetkin Agricultural Machinery Application Workshop, Department of Agricultural Machinery and Technologies Engineering, Faculty of Agriculture, Selçuk University, Konya, Turkey.

The test facility was specifically designed to reproduce the hydraulic conditions encountered in agricultural groundwater pumping systems. By controlling flow rate, submergence depth, drawdown level, discharge pressure, and pump operating conditions, various cavitation scenarios were experimentally generated. This controlled environment enabled the collection of reliable data representing both normal operating conditions and different stages of cavitation development.

**Figure 8:**
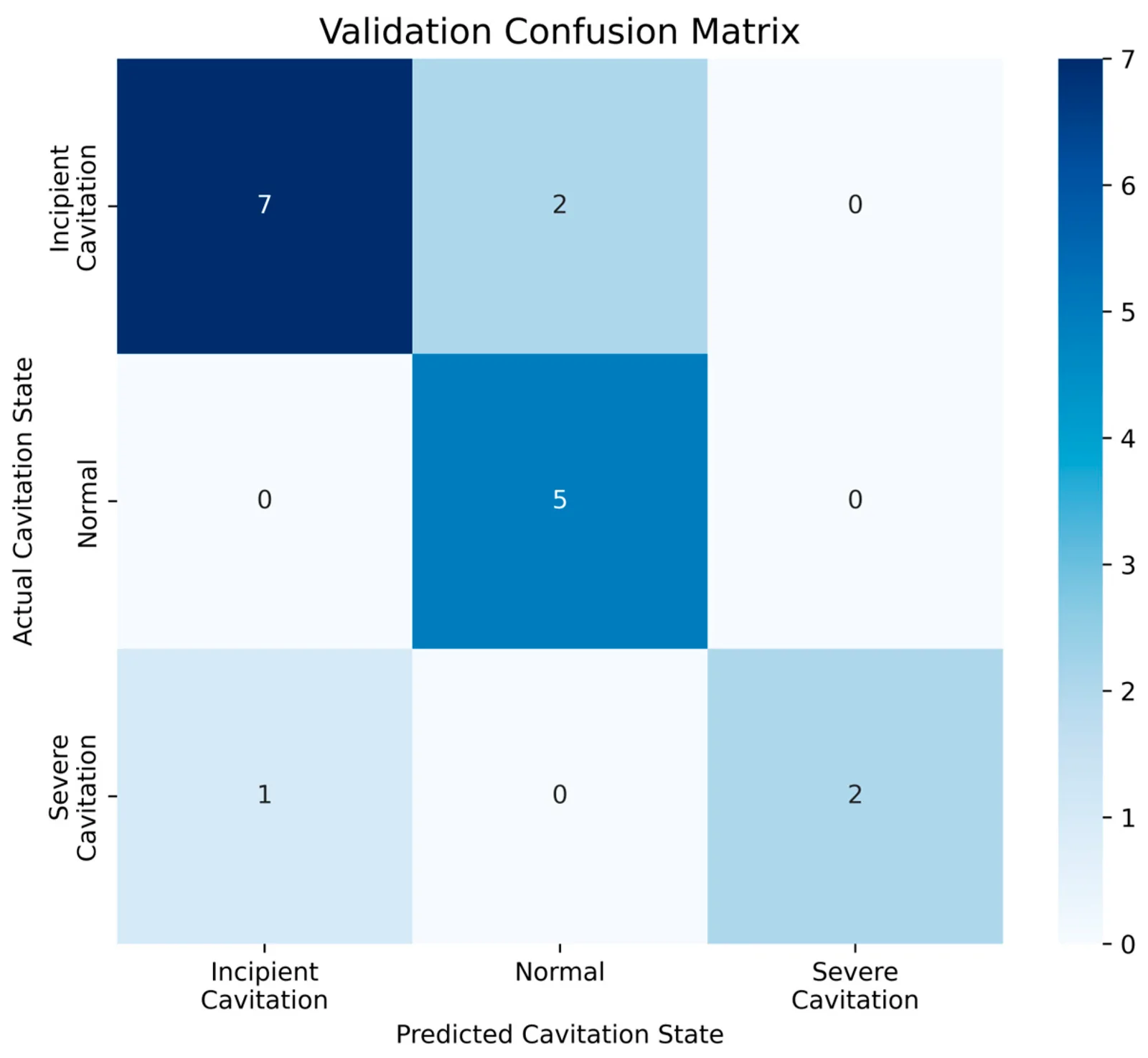
Confusion matrix obtained from the Extra Tree classifier using 17 experimental operating conditions.

The physical validation was conducted using 17 unseen operating conditions contained in the independent test dataset representing different hydraulic states, including Normal, Incipient Cavitation, and Severe Cavitation conditions. The corresponding confusion matrix is presented in Figure 8.

As shown in Figure 8, the classifier correctly identified 14 of the 17 test cases, corresponding to an overall classification accuracy of 82.4%. Among the 9 Incipient Cavitation cases, 7 were correctly classified, while two were incorrectly assigned to the Normal class. All 5 Normal operating conditions were successfully identified without any misclassification. For the Severe Cavitation category, two of the three cases were correctly classified, whereas one case was classified as Incipient Cavitation.

From a physical perspective, the obtained results are consistent with the progressive nature of cavitation development. Cavitation does not occur instantaneously but evolves gradually as hydraulic conditions deteriorate. Consequently, operating conditions associated with Incipient and Severe Cavitation may exhibit similar hydraulic and acoustic characteristics, which explains the limited number of misclassifications observed between these neighboring classes.

An important observation is that no Normal operating condition was classified as Severe Cavitation and no Severe Cavitation condition was classified as Normal operation. This finding demonstrates that the proposed framework can reliably distinguish healthy pump operation from advanced cavitation conditions. Such capability is particularly important for agricultural pumping systems, where severe cavitation may result in efficiency losses, excessive noise generation, mechanical wear, and reduced equipment lifetime.

Overall, the physical validation results confirm that the proposed machine learning framework not only achieves satisfactory statistical performance but also produces predictions that are consistent with the physical behavior of cavitation observed under real operating conditions.

The proposed framework is intended to estimate cavitation conditions using hydraulic and operational variables that are commonly available in pumping systems, rather than relying on continuous acoustic monitoring. Although acoustic measurements were employed during the experimental labeling stage, they are not required during practical operation of the trained model. Consequently, the proposed approach can support cavitation monitoring in applications where acoustic sensing is impractical due to environmental noise, installation constraints, or sensor availability.

## 6. Conclusions

This study presented a machine learning-based framework for the detection of cavitation conditions in submersible deep well pumps using experimental multi-sensor data collected under controlled operating conditions. The proposed approach utilized hydraulic and operational parameters, including flow rate, submergence depth, drawdown, power consumption, and discharge pressure, to automatically classify pump operating conditions into Normal, Incipient Cavitation, and Severe Cavitation categories. The experimental results demonstrated that machine learning techniques can successfully identify cavitation conditions using commonly measured pump parameters. Among the evaluated algorithms, the Extra Tree classifier achieved the best overall performance with an accuracy of 82.4%, precision of 85.0%, recall of 82.4%, and F1-score of 82.2%. Furthermore, the confusion matrix analysis showed that no direct misclassification occurred between Normal and Severe Cavitation conditions, indicating that the developed framework is capable of reliably distinguishing healthy pump operation from advanced cavitation states.

The correlation and feature importance analyses revealed that power consumption and submergence depth were the most influential parameters for cavitation detection. These findings are consistent with the physical mechanisms governing cavitation formation in deep well pumping systems and provide additional confidence regarding the reliability of the developed models.

From a computational perspective, all evaluated machine learning algorithms exhibited very short training times, with an average training duration of only 0.052 s during model development. More importantly, the selected tree-based classifiers perform inference through simple decision rules using a limited number of input features, making the proposed framework computationally lightweight and suitable for real-time monitoring applications. These characteristics indicate that the framework is a promising candidate for future implementation on resource-constrained edge devices commonly used in agricultural irrigation systems.

Overall, the results demonstrate that machine learning-based cavitation detection offers a practical and effective solution for improving the operational reliability of agricultural pumping systems. By enabling early identification of cavitation-related conditions, the proposed framework has the potential to reduce maintenance costs, prevent performance degradation, and extend pump service life. Unlike acoustic threshold-based approaches, the trained machine learning model does not require continuous acoustic measurements during operation. Instead, cavitation conditions are inferred from hydraulic and operational parameters that are commonly monitored in pumping systems. Since acoustic measurements were used only during the offline dataset labeling stage, the proposed framework provides a practical alternative for applications where continuous acoustic monitoring is impractical or unavailable.

One limitation of this study is that the dataset was collected from a single experimental setup and involves a limited number of operating conditions. Although controlled experiments ensure data reliability, this may limit the generalizability of the model across different pump types, operating environments, and field conditions. In particular, the independent test dataset consisted of a limited number of operating conditions. Therefore, the reported results should be interpreted as an experimental validation of the proposed methodology rather than definitive evidence of its generalization capability. Therefore, future studies may focus on expanding the dataset using different pump types, operating conditions, and environmental scenarios to further assess the robustness and generalizability of the proposed framework. Also, since the cavitation classification thresholds were derived from observations in the experimental specific setup, future studies may incorporate multi-physics indicators such as vibration signals, pressure pulsations, and high-speed visualization to establish more robust and generalized cavitation labeling strategies. In addition, although the proposed framework achieved satisfactory performance, the classification accuracy indicates that there is still room for improvement, particularly in distinguishing between Incipient and Severe cavitation stages.

Although the proposed framework demonstrated satisfactory classification performance under stratified cross-validation and independent test conditions, its generalization capability under completely unseen operating conditions (e.g., unseen flow rates or water levels) was not investigated in the present study. Future work will consider leave-one-flow-rate and leave-one-water-level validation strategies to evaluate the robustness and transferability of the proposed approach under previously unseen hydraulic operating conditions. Hence, future work may explore advanced optimization techniques, hyperparameter tuning, and hybrid or ensemble learning strategies to further improve prediction accuracy. Moreover, while the selected features provide valuable information for cavitation detection, the inclusion of additional sensing modalities such as vibration and frequency-domain signal features may further enhance model performance. Finally, although the proposed framework was specifically designed to satisfy the computational requirements of edge computing applications, experimental validation on embedded hardware platforms (e.g., Raspberry Pi or microcontroller-based systems) has not yet been performed. Therefore, future work will focus on implementing and experimentally evaluating the proposed framework on representative embedded edge platforms under real operating conditions.

## Figures and Tables

**Table 1:** Hyperparameter settings of the evaluated machine learning models.

Model	Hyperparameter Settings
ExtraTree	criterion = gini, splitter = random, max_depth = None, random_state = 42
RandomForest	n_estimators = 200, criterion = gini, max_depth = None, random_state = 42
GradientBoosting	learning_rate = 0.1, n_estimators = 100, random_state = 42
Bagging	n_estimators = 200, random_state = 42
DecisionTree	criterion = gini, splitter = random, max_depth = None, random_state = 42
ExtraTrees	n_estimators = 200, criterion = gini, max_depth = None, random_state = 42
LogisticRegression	solver = lbfgs, penalty = l2, max_iter = 1000
AdaBoost	n_estimators = 50, learning_rate = 1.0, random_state = 42
KNN	n_neighbors = 5, weights = uniform, metric = minkowski
HistGradientBoosting	learning_rate = 0.1, max_iter = 100, random_state = 42
NaiveBayes	GaussianNB (default settings)
SVM (RBF)	kernel = rbf, C = 1.0, gamma = scale, decision_function_shape = ovr
MLP (ANN)	hidden_layer_sizes = (100,), activation = relu, solver = adam, alpha = 0.0001, learning_rate = constant, max_iter = 200, random_state = 42

**Table 2:** Performance comparison of the evaluated machine learning models.

Model	CV Accuracy	Accuracy	Precision	Recall	F1-Score
ExtraTree	0.848	0.824	0.850	0.824	0.822
RandomForest	0.818	0.765	0.765	0.765	0.765
GradientBoosting	0.804	0.706	0.709	0.706	0.704
Bagging	0.793	0.765	0.765	0.765	0.765
DecisionTree	0.781	0.765	0.765	0.765	0.765
SVM (RBF)	0.780	0.763	0.763	0.763	0.762
ExtraTrees	0.769	0.765	0.765	0.765	0.765
LogisticRegression	0.696	0.706	0.735	0.706	0.695
AdaBoost	0.684	0.412	0.231	0.412	0.281
KNN	0.671	0.647	0.698	0.647	0.620
MLP (ANN)	0.658	0.476	0.478	0.471	0.451
HistGradientBoosting	0.657	0.471	0.446	0.471	0.450
NaiveBayes	0.587	0.529	0.559	0.529	0.515

**Table 3:** Comparison of representative machine learning-based cavitation detection studies.

Refs.	Input Feature	Method	Accuracy	Remarks
[38]	Vibration	SVM	99.4%	Vibration-based monitoring
[39]	Vibration	ANN	High (>95%)	High-speed photography used for labeling
[40]	Vibration	CEEMDAN + BOA + SVM	96.8%	Multi-channel vibration
[41]	Vibration	ANN	99.86%	Hybrid ANN–DT framework
This Study	Hydraulic + operational parameters	Extra Tree	82.4%	No vibration or acoustic signal used as model input

**Table 4:** Performance comparison of the proposed framework using different input configurations.

Input Configuration	CV Accuracy	Test Accuracy
With Drawdown	0.848	0.824
Without Drawdown	0.817	0.740

**Table 5:** Class-wise performance metrics of the best-performing Extra Tree classifier.

Model	Precision	Recall	F1-Score
Normal	0.91	0.90	0.90
Incipient	0.77	0.73	0.75
Severe	0.89	0.91	0.90

**Table 6:** Training times of the evaluated machine learning models.

Model	Training Time (s)
ExtraTree	0.001
DecisionTree	0.001
RandomForest	0.113
ExtraTrees	0.086
Bagging	0.153
GradientBoosting	0.103
LogisticRegression	0.002
KNN	0.002
NaiveBayes	0.002
HistGradientBoosting	0.074
AdaBoost	0.034
**Average**	**0.052**

## Data Availability

The experimental dataset used in this study was generated using a custom-built laboratory test platform developed by the authors. Because the study forms part of an ongoing research program, the complete dataset is not currently deposited in a public repository. However, the dataset is available from the corresponding author upon reasonable request.
